# Jointly They Edit: Examining the Impact of Community Identification on Political Interaction in Wikipedia

**DOI:** 10.1371/journal.pone.0060584

**Published:** 2013-04-03

**Authors:** Jessica J. Neff, David Laniado, Karolin E. Kappler, Yana Volkovich, Pablo Aragón, Andreas Kaltenbrunner

**Affiliations:** 1 Annenberg School for Communication & Journalism, University of Southern California, Los Angeles, California, United States of America; 2 Barcelona Media–Innovation Centre, Barcelona, Spain; Université de Lausanne, Switzerland

## Abstract

**Background:**

In their 2005 study, Adamic and Glance coined the memorable phrase ‘divided they blog’, referring to a trend of cyberbalkanization in the political blogosphere, with liberal and conservative blogs tending to link to other blogs with a similar political slant, and not to one another. As political discussion and activity increasingly moves online, the power of framing political discourses is shifting from mass media to social media.

**Methodology/Principal Findings:**

Continued examination of political interactions online is critical, and we extend this line of research by examining the activities of political users within the Wikipedia community. First, we examined how users in Wikipedia choose to display their political affiliation. Next, we analyzed the patterns of cross-party interaction and community participation among those users proclaiming a political affiliation. In contrast to previous analyses of other social media, we did not find strong trends indicating a preference to interact with members of the same political party within the Wikipedia community.

**Conclusions/Significance:**

Our results indicate that users who proclaim their political affiliation within the community tend to proclaim their identity as a ‘Wikipedian’ even more loudly. It seems that the shared identity of ‘being Wikipedian’ may be strong enough to triumph over other potentially divisive facets of personal identity, such as political affiliation.

## Introduction

Online media have become an increasingly important source of political information in recent years. This trend emerged most notably in the 2004 U.S. presidential campaign. For the first time, political blogs served as a prominent information source regarding the campaign and candidates [1], and candidates themselves began to leverage the power of online platforms to organize and raise money [2]. The trend of utilizing online platforms for political purposes, and in particular for the dissemination of information, has continued to grow in recent years. People consult political blogs, gain information about politicians, legislation and emerging social movements through their Facebook pages, and turn to online resources such as Wikipedia for up-to-date information on political issues [3]. Given the increasing prominence of the Web, and social sites in particular, as sources of political information, it is crucial to take a closer look at the patterns of interaction and discourse that members of different political parties have around information online, because they may have important consequences for the accuracy and neutrality of political information provided online.

### Political Interaction Online

Much of the research examining political interaction online has provided support for a trend of polarization. One of the seminal studies in this area was Adamic and Glance's [1] examination of the political blogosphere. They examined linking behavior among political blogs in the months leading up to the 2004 U.S. Presidential election, and found that conservative and liberal political blogs primarily link to other blogs with their same political orientation and exhibit far fewer links to blogs that do not fall within their own political community. Using a different dataset and a different methodology, Ackland [4] replicated Adamic and Glance's findings [1], providing additional evidence for polarization in the political blogosphere.

In a similar study Hargittai, Gallo, and Kane [5] provided further support for this trend. Their examination of linkages among political blogs also revealed a tendency for blogs to link with blogs that are ideologically similar; however, they also found evidence for cross-ideological linkages. Qualitative analysis of these linkages indicated that the vast majority of these links are used in the context of “straw-man” arguments, and therefore are not indicative of true cross-party dialogue. Blog readership also follows similar patterns of fragmentation. For example, Lawrence, Sides & Farrell [6] found that people tend to read blogs that reinforce, rather than challenge, their political beliefs. Taken together, these studies provide strong support for the trend towards fragmentation and polarization on the political blogosphere across party lines.

Political polarization has also been observed in other online contexts. For example, Feller, Kuhnert, Sprenger, and Welpe [7] considered patterns of interaction among political users on Twitter. They analyzed a sample of 2,500 German Twitter users. From this large sample they generated a subsample of 759 political users. In line with Adamic and Glance's results [1], they also found patterns of preferential interaction based on political party. Users were more likely to be connected to other users who shared the same party affiliation. When linking was seen across parties, it was more frequent among member of parties that were more ideologically similar. Research has revealed similar findings for political Twitter users in the U.S.. Conover et al. [8] analyzed tweets containing politically valenced hashtags in the six weeks leading up to the 2010 midterm Congressional elections. They found strong evidence of political polarization in the network of retweets, with users more likely to retweet users with whom they share the same political ideology. Similar results have been found in multiple party environments, such as the Twitter-campaign leading up to the Spanish national elections in 2011 [9].

In contrast to the findings from the blogosphere and Twitter, research on interactions in political newsgroups does not paint a clear picture of polarized interactions. Kelly, Fisher, and Smith [10] analyzed the discussion networks of members of political newsgroups on Usenet and found a great deal of cross-party interaction, indicating that these newsgroups were spaces for “debate and deliberation”, as opposed to “ideological echo chambers” [10]. They also identified distinct types of users. The vast majority of users were what they termed ‘fighters’, that is, they exhibited a preference to interact with members of opposing political parties and were less likely to interact with members of their own political party. They also identified a class of users they called ‘friendlies’, who only engage with allies (or same party users), and ignore users with opposing party affiliation and viewpoints. However, these users were much less common than the fighters. In another study of patterns of interaction in online discussion groups, Wojcieszak and Mutz [11] also show support for cross-party engagement in online discussion groups.

The aforementioned studies have provided an unclear and somewhat conflictive picture of what cross-party political interaction looks like online. In some contexts (e.g. the blogosphere, Twitter) interactions that cross ideological divides are rare. However, in other settings (e.g. online discussion boards), there is evidence for higher rates of interactions across party lines. Taken together these finding indicate that the degree of interaction and engagement with politically dissimilar others varies across contexts.

Understanding when and why people engage in political debate and discussion online is important. The degree of interaction or insularity of political groups producing political information online can have important consequences for information consumers, because it may influence the extent to which issues are presented in a biased or neutral way. The present research seeks to address this issue, and to shed light on patterns of political interaction within the Wikipedia community.

### Political Interaction in Wikipedia

Wikipedia, currently the 6th most trafficked site in the world [12], is arguably one of the most important information sources on the Web. In January 2012 it received 482 million global unique visitors [13]. For many people, Wikipedia is the first site they visit when they want to familiarize themselves with a new topic. A recent poll revealed that as of May 2010, 53% of U.S. users of the Internet sought out information in Wikipedia. A web search yields a link to a Wikipedia entry among the top three search results almost 90% of the time [14]. Past research has revealed that Wikipedia entries on topics from a variety of different disciplines [15], including politics [16], are extremely accurate.

Wikipedia is unique when compared to other online references. In the world of online information there is professional content, some of which aims for a neutral stance and some of which has a self-proclaimed bias, and there is user generated content (UGC), which, at its core, reflects the beliefs and ideologies of those who create it. Wikipedia is built entirely on UGC; however at the same time explicitly espouses neutrality. One of the fundamental rules of the community is that all articles must be edited from a neutral point of view (NPOV). In Wikipedia, neutrality “means carefully and critically analyzing a variety of reliable sources and then attempting to convey to the reader the information contained in them clearly and accurately. Wikipedia aims to “describe disputes, but not engage in them” [17]. This marks a difference with respect to other online references, such as Conservapedia [18], which was created in opposition to Wikipedia and explicitly espouses a conservative point of view.

While neutrality is a fundamental principle of Wikipedia, members have a diverse array of beliefs and values. Therefore, it is particularly interesting to examine how diverse, and at times contentious, groups interact on the site. How is it that these people come together to create neutral content? Is there fragmentation, as we see in the blogosphere and on Twitter, or is there interaction and debate like we see in communities such as Usenet?

Extant research has generally failed to consider interactions among members of different political parties on Wikipedia. However, there are a couple of exceptions. One is a recent study that examined edits made to the 2012 Republican presidential candidate Mitt Romney's Wikipedia page [19]. Some of the most edited topics on the page were those related to controversies surrounding Romney, which were frequently invoked in partisan debates. Findings also indicated that the peak in the number of edits made to the page coincided with the Florida Presidential primaries. As a potential explanation for the timing of this peak, the researchers suggest that perhaps users are making edits in an effort to influence public opinion. A confirmation of the importance of partisan disputes in articles about politics is provided in another work [20], where the article on Barack Obama was taken as a case study for understanding temporal patterns of activity in Wikipedia. Peaks in both editing and discussion activity were observed in conjunction with elections and other relevant offline events. These studies provide some indication that there may be partisan conflicts taking place among Wikipedia users. However, they only looked at editing behavior in general and did not examine the political affiliations of individual users. In the present research we seek to provide a more in-depth look at politics in Wikipedia by examining patterns of interaction among self-proclaimed Republican and Democratic users through the lens of social identity theory.

### Social Identity Theory

Social identity theory [21] provides a theoretical framework for understanding patterns of cross-party interaction. This theory and the related self-categorization theory [22], [23] address how identification and categorization influence intergroup interactions. The central thrust of these theories pertains to the existence of multiple, socially defined ‘selves’. They maintain that we do not have a single, static self, but rather that we have a variety of different self categorizations that may become salient depending on what context we are in [23]. These categorizations may be either personal identities or social identities. A person can have any number of personal and social identities. Spears and Lea [24] provide the following description of the self-categories available to individuals:

“…self categories can be ordered in terms of a hierarchy of abstraction and include personal identities (which distinguish the person from other individuals or in-group members) and social identities (which define them as similar to other in-group members and different from out-groups on relevant dimensions). In sum, the salient self category is highly flexible and context dependent.” [24]

Social identity can be derived from membership in a formal group (e.g. a soccer team), but can also be derived from more abstract groups or categorizations (e.g. race, gender). Tajfel and Turner [20] provide a broad-based description of groups, defining a group as “a collection of individuals who perceive themselves to be members of the same social category, share some emotional involvements in this common definition of themselves, and achieve some degree of social consensus about the evaluation of their group and of their membership in it” [21]. Social identification results in a sort of “us” versus “them” dynamic, with individuals treating in-group members preferentially and discriminating against out-group members.

### Social Identity and Party Affiliation

Social identity theory has been applied to the domain of politics, and research has demonstrated that people can develop social identities stemming from political party affiliation [25]. Political identity has been offered as a theoretical explanation for the strong partisan tensions that emerge, for example, between the U.S. Republican and Democratic parties. Identification with a political party can lead an individual to selectively attend to information that supports his or her own party, while ignoring information that supports the other party [26]. In one of the first studies of social identity in the context of U.S. politics, Greene [26] found that the strength of an individual's party identification was a significant predictor of ratings of in-party and out-party members. Individuals with strong party identification had more favorable ratings of in-party members and less favorable ratings of out-party members, in contrast to individuals who exhibited weaker party affiliation.

A later study extended these findings [27], and revealed that strength of party identification is also significantly related to likelihood of engaging in partisan activities (e.g. making financial contribution to a campaign, attending a campaign rally, etc.) and voting for the party in elections. Fowler and Kam [28] also found that strength of political identification is linked to political participation. Taken together, these findings provide strong support for the claim that social identity can be derived from political party membership, and that such identification can have an important impact on perceptions of, and interactions with, members of other political parties. This insight helps us to make sense of findings from previous research on political interaction online. Individuals with strong party affiliations (e.g. political bloggers, activists who tweet) will likely prefer to interact with members of their same party, and will view same party members in a more positive light.

### Social Identity in Online Communities

Membership in an online community may also be a source of social identity. Recent theoretical work by Ren, Kraut, and Kiesler [28] has explored this phenomenon in greater depth. Ren et al. argue that individuals can develop attachments to online communities based on a common identity (an attachment to the community at large) and common bonds (an attachment to individual community members). Attachments based on common identity are most relevant for the present discussion. The authors note that, “in general, common identity in the online context implies that members feel a commitment to the online community's purpose or topic” [28].

One source of common identity in online communities is task interdependence. When community members are working together to accomplish a joint task, this can foster a sense of shared identity [29]. Wikipedia is an example of such a community. A diverse group of people comes together to create a shared good–a collaboratively authored encyclopedia. Another source of common identity is sense of community, a concept that was originally proposed in the context of offline communities [30], [31], which has since been extended to the virtual domain [32]. Sense of community is “a feeling that members have of belonging, a feeling that members matter to one another and to the group, and a shared faith the members' needs will be met through their commitment to be together” [30]. The sense of community that users feel in Wikipedia may also drive users' identification with the community. Rafaeli and Ariel [33] have posited that the sense of community that users derive from Wikipedia may be one of their primary motivations for participation.

A study conducted by Bryant, Forte and Bruckman [34] provides evidence for the presence of a sense of a community within Wikipedia. They described the process by which newcomers move from the periphery of the community to taking on more active and central roles. As users become more involved in the site, a transformation takes place in their identity, as they come to view themselves as *members of the tribe* and gain awareness of social roles in the community. This transformation is accompanied by a shift in activity, as members move “from a local focus on individual articles to a concern for the quality of the Wikipedia content as a whole and the health of the community” [34].

It is important to note that the presence of a Wikipedian identity does not preclude the existence of other identities. Depending on the activities that a community member is performing the Wikipedian identity may or may not be activated. For example, when making edits to a particular page, the social identity a user has that is associated with that particular topic (e.g. “Republican” while editing the George W. Bush page, “soccer player” while editing the Lionel Messi page, or “mother” while editing the page on Childhood Development) may become activated.

### Research questions

The preceding discussion has reviewed the formation of social identity, and the influence that identity can have on intergroup dynamics. Individuals may have multiple social identities that become more or less salient depending on the social context. Members of the Wikipedia community who publicly declare their political party affiliation represent a minority of users. However, the fact that these users choose to call attention to this aspect of their identity is noteworthy. Therefore, it seems possible that either the social identity of party affiliation or of being Wikipedian could be activated in the context of this community. In the present research we examine user practices of representation and identity and examine patterns of cross-party interaction. In light of the preceding review, we pose the following research questions:

- RQ1: What are the identity and representation practices of users who claim their affiliation to a party within the Wikipedia community?
- RQ2: Do we see a division in patterns of participation along party lines?
- RQ3: Do users exhibit a preference for interacting with members of their same political party?
- RQ4: Does political affiliation of users affect the amount of conflict in discussions?

## Materials and Methods

### Overview

We conducted a mixed-methods analysis of patterns of activity, interaction, and identity representation practices among 1,390 members of the Wikipedia community who explicitly proclaimed their political affiliation as either a Republican or Democrat. In order to determine user political affiliation, user pages were examined. Content analysis was used to evaluate user representation practices in user profiles, and to categorize and thematically group the most edited articles by members of each party. Social network analysis was used to explore the research questions regarding patterns of interactions. Finally, content analysis was used to examine differences to conflict across and within parties, and to evaluate user representation practices in user profiles.

### User selection

**Figure 1:**
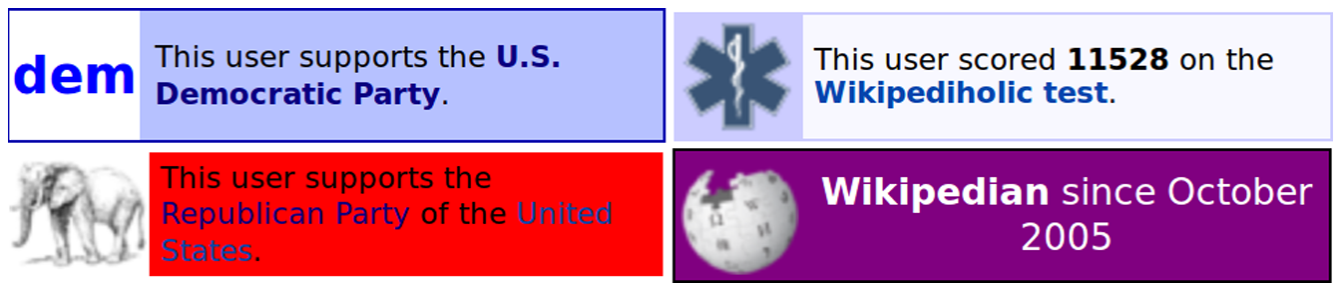
Examples of Userboxes.

On personal user pages users have the ability to display userboxes. A userbox is “a small colored box designed to appear only on a Wikipedian's user page as a communicative notice about the user, in order to directly (or indirectly) help Wikipedians collaborate more effectively on articles” [35]. Userboxes are customizable, and a user can choose to include any information she would like. See Figure 1 for an example of userboxes. In order to select Republican and Democratic users, userboxes that identified a user as a Republican or Democrat were manually identified and then collected automatically. Users were included as members of a party if they had a box on their user page that expressed explicit support for a party (e.g. “This user supports the GOP”, “This user supports the Democratic Party”) and/or a userbox that expressed support for a particular political candidate (e.g. “This user supports Barack Obama for President” or “This user supports John McCain”). In order to be able to identify different kinds of userboxes and templates, we searched in the “User” namespace for links to the articles of the Democratic and Republican parties and of their major leaders. Additionally, we searched for specific sentence patterns in namespace “User”, such as “This user supports the * party”. Using this method to identify users resulted in a sample of 863 Democrats and 527 Republicans, which should correspond to nearly all users who disclosed their support for one of the two major U.S. political parties in a userbox.

### Identity analysis

Members of the Wikipedia community have the option of creating a customized user page. Pages can be personalized to reflect the preferences and interests of the individual users, and one of the primary ways that users personalize their pages is through the use of userboxes, which were described above. A qualitative analysis of the userboxes of a randomly selected sample of fifty Democratic and fifty Republican users was conducted.

First, the overall number of userboxes for each user was tallied. Next, the number of political party boxes that a user listed on his or her page was tallied. A box was counted as a party box if it explicitly expressed support for, or membership in, the Republican or Democratic Party. The number of politically oriented userboxes that were *not* political party boxes was also tallied. Politically oriented userboxes were coded as “conservative”, “liberal”, or “other”. Boxes coded as conservative were those that expressed what is generally considered a conservative ideology. Examples include, “This user is pro-life”, “This user supports LEGAL immigration”, and “this user thinks the global warming issue has been immensely exaggerated”. Boxes coded as liberal were those that expressed what is generally considered a liberal ideology. Examples include, “This user supports the legalization of same-sex marriage”, “This user is pro-choice”, and “This user supports immigration and the right to travel freely upon the planet we share”. Issues coded as “other” were those that dealt with some political issue, but are not generally assigned to a particular political ideology. Examples include, “This user wants ZERO net carbon emission from human activity”, “This user is against monarchy”, and “This user condemns and opposes Srebrenica Genocide denial”.

Content analysis of user walls was performed on a randomly selected subsample of 100 user pages (50 Republicans, 50 Democrats). Intercoder reliability was assessed using Holsti's reliability score [36], which measures the percent agreement between two coders ratings. The obtained coefficient of.84 was acceptable.

To verify that these subsamples represent the original samples, t-tests have been performed comparing the registration time and the logarithm of the number of comments and edits plus one The logarithm was used to account for the heavy tailed nature of these distributions. Details on the corresponding distributions are given in the following section. In all cases and for both groups of partisan users, the null hypothesis that sample and subsample are extracted from the same distributions cannot be rejected.

### Activity and interaction data extraction

**Figure 2:**
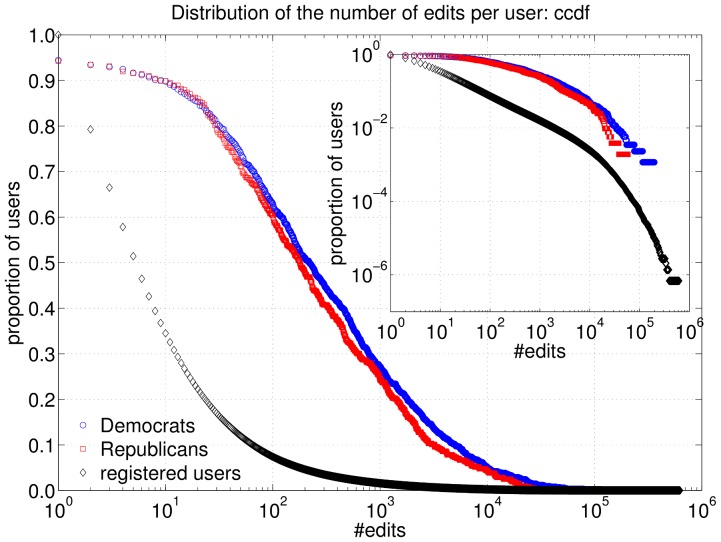
Complementary cumulative distribution of the number of edits per user, for Democrats (blue circles), Republicans (red squares) and all Wikipedia users (black diamonds). Inset shows the same data in log-log scale. In the case of all users only users with at least one edit are considered.

Activity and interaction data came from a complete dump of the English Wikipedia, dated March 12^th^ 2010. First, we considered edit activity. We counted all edits made by users in our sample to each Wikipedia article. Figure 2 shows the complementary cumulative distribution of the number of edits per user, broken down by party: the distribution for Democrats is depicted in blue circles, for Republicans in red squares. The two curves are very similar, with about 75% of the users having more than 50 edits, and about 25% of users with over 1000 edits. The major difference is that the most active users in our sample, reaching the order of 100 thousand edits, are Democrats. We also compare the two groups of partisan users with the entire set of registered users who had made at least one edit (depicted in black diamonds in Figure 2). We clearly observe that the set of partisan users contains a much larger proportion of active users. The inset depicts the same curves in log-log scale, showing a heavy-tailed distribution, but not the typical straight lines observed for power law distributions. A double Pareto distribution (a combination of two power-laws with different exponents) seems to be a better explanation for the data. This becomes especially evident in the distribution of all registered users and may be explained by the large variations in the registration time of the users on Wikipedia [37].

We also extracted comments written by users in talk pages, i.e. special wiki pages devoted to communication among editors (also included in the above mentioned complete dump). We considered both *article talk pages*, where users can discuss issues concerning the corresponding articles, and *user talk pages* (aka *user walls*), which are used by editors to exchange personal messages. Data were obtained by parsing the source text of talk pages and identifying user signatures and comment indentation to reconstruct the thread structure, as described by Laniado, Tasso, Volkovich and Kaltenbrunner [38].

**Figure 3:**
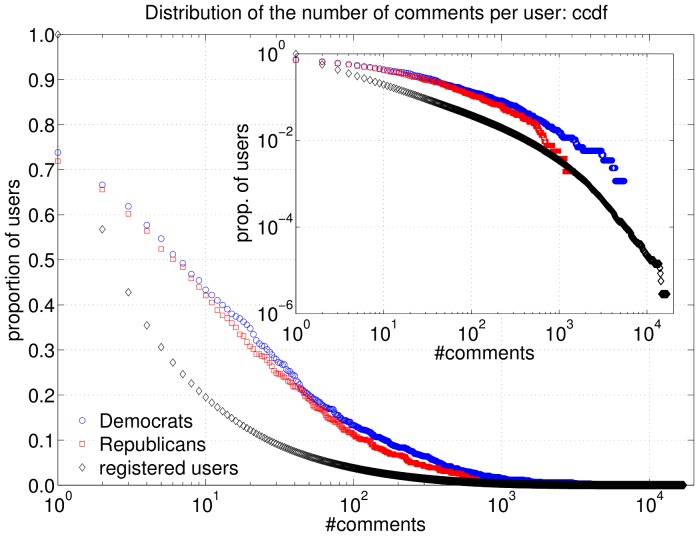
Complementary cumulative distribution of the number of comments per user, for Democrats (blue circles), Republicans (red squares) and all Wikipedia users (black diamonds). Inset shows the same data in log-log scale. In the case of all users, only users with at least one comment are considered.

The distribution of the number of comments per user in article talk pages (Figure 3) shows that within both parties around 50% of users wrote more than 5 comments and 20% wrote more than 50. Again, we find similar distributions for Democrats and Republicans, with the most active users among Democrats. The figure also shows that both groups contain a larger proportion of users who have made at least a few comments than the Wikipedia community in general.

**Figure 4:**
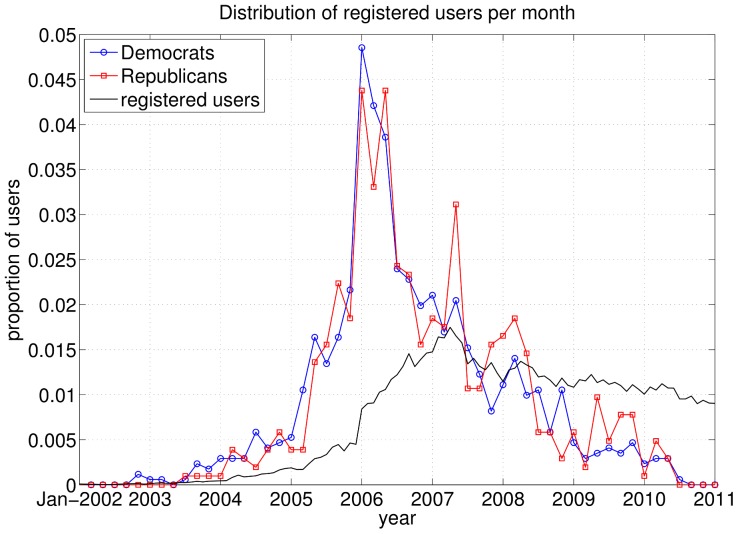
Distribution of the registration time of the Wikipedians. Note that the depicted values are per month for the set of all registered users (black curve with diamonds) while for the two groups of partisan users values are averaged over two month periods to filter out fluctuations due to the smaller amount of users in these groups.

A further comparison between the partisan users and the overall Wikipedia users is made in Figure 4, depicting the proportion of registered users per time interval. The distribution of the registration dates for all users is based on data extracted from [39]. We observe that the two groups of partisan users have nearly the same distribution and are composed of more veteran users than the Wikipedia in general. This helps to explain their larger commitment towards the site and their more active participation.

To analyze patterns of communication among our set of users, we identified two networks of interactions based on messages written by the users in talk pages. From article talk pages we extracted a *reply network*, by establishing a connection from user A to user B if user A has replied to a comment written by user B. From *user walls* we extracted a *wall network* by connecting user A to user B if user A has left a message on user B's personal talk page. Basic statistics of the two resulting directed unweighted networks are given in Table 1. It should be noted that the networks do not include all users in our sample, but only the ones who have interacted with other users in the sample. As a result, the reply network includes only 270 users (161 Democrats and 109 Republicans), while the wall network includes 435 users (258 Democrats and 176 Republicans). For both networks, we also obtained a weighted version by assigning to each edge the corresponding number of messages. For example, the edge from user A to user B in the reply network has weight 3 if user A has replied to three comments by user B.

**Table 1:** Basics statistics of the two interaction networks.

	Nodes	Dem	Rep	Edges	Giant comp.	Reciprocity	Clustering coefficient		Average distance
Reply	270	161	109	430 (854)	83.9%	0.28	0.040	4.75	
Wall	434	258	176	997 (2619)	95.6%	0.29	0.074	4.00	

Basic statistics of the two networks based on replies in article talk pages (*Reply*) and on messages written on personal talk pages (*Wall*). Total number of *nodes*, broken into Democrats (*Dem*) and Republicans (*Rep*), and of *edges* (in parenthesis, the sum of the edge weights). Metrics for the two unweighted directed networks: size of the *giant component* (weakly connected), *reciprocity*, global *clustering coefficient* and *average distance* between nodes.

### Most edited articles

Lists of the most frequently edited articles among Democrats, Republicans, and all Wikipedia editors were generated. The number of articles shared by various groups was calculated. The articles were also coded according to topics. Articles were coded as “Political” (relating to a political issue or a politician, e.g. United States Presidential Election, 2008; George Bush) or “Not Political” (e.g. Britney Spears, 2008 Summer Olympics). Political pages were further coded as “Conservative” (related to a conservative politician, commentator, or issue, e.g. Rush Limbaugh), “Liberal” (related to a liberal politician, commentator, or issue, e.g. Al Gore), or “Neutral” (political in nature, but not partisan, e.g. European Union, September 11 attacks).

### Cross-party interactions

To assess whether there is a preference for interaction among editors belonging to the same party, or to different parties, we studied the mixing coefficient of the networks, and we performed a shuffle test in order to assess statistical significance.

We first extracted from each network a matrix representing how many connections (based on comments) are directed from a Democrat to a Democrat, from a Democrat to a Republican, and so forth. We then normalized these matrices and computed the mixing coefficient as the preference for inter-party or for intra-party interaction, according to Newman [40]. To assess statistical significance, we contrasted the results with a sample of randomized equivalents of the networks. More specifically, keeping the users fixed, (both in terms of their party affiliations and their numbers of in-coming and out-going links), we randomized the links between them, thereby generating a sample of 100 networks where the users have the same number of connection as in the original network but are connected to different users. We computed the average value *r*_rand_ of the mixing coefficient in these networks, and the standard deviation σ*_rand_*; finally we computed the *Z-score* as the difference between the value observed in the real network and the average over the randomized networks, in units of the standard deviation (Z-score = (r−*r*_rand_)/σ*_rand_*). High positive values of Z indicate a preference for inter-party interactions, while high negative values indicate a preference for intra-party interactions. Results low in absolute values (|Z|<2) correspond to neutral mixing, i.e. no statistically significant preference for either inter- or intra-party interaction [41].

### Conflict

We examined discussion thread conflict in order to assess whether or not users exhibit different interaction styles with same party members versus different party members. All discussion threads on article talk pages that included at least two Democrats and no Republicans (572), at least two Republicans and no Democrats (147), or at least one Democrat and one Republican (583) from our sample were extracted from the data set. From this sample, we then selected all threads related to articles that dealt with political or other potentially controversial topics. Examples include “War on Terrorism”, “Mike Huckabee”, “Eliot Spitzer”, and “Mahmoud Ahmadinejad”. These threads were then coded for whether or not they were conflictual, and for whether or not the conflict was political in nature. This resulted in 144 threads with at least one Democrat and one Republican, 130 threads with two or more Democrats, and 71 threads with two or more Republicans. Holsti's reliability score [36] was used to assess intercoder reliability. At.82, the coefficient was acceptable.

## Results

### Identity analysis

First, we tested to see if there were differences in the average number of userboxes listed on the profiles of Republicans and Democrats. There was no significant difference (p = 0.3). Unsurprisingly, Republicans (M = 3.06, SD = 5.37) had a significantly higher number of conservatively valenced user boxes than Democrats (M = 0.08, SD = 0.44) (*t* = 4, *p*<0.001), while Democrats (M = 2.51, SD = 3.47) had a significantly higher number of liberally valenced user boxes than Republicans (M = 0.27, SD = 0.60) (*t* = 4, *p*<0.001). Next we looked to see if there were any differences in number of Wikipedia related user boxes listed on the user pages of Democrats and Republicans, but we did not find any (p = 0.07). Finally, we examined differences between the number of “Wikipedia” boxes and “Party” boxes for members of each party. Democrats had significantly more “Wikipedia” boxes (M = 4.70, SD = 3.71) than “Party” boxes (M = 1.44, SD = 1.34), *t* = 3.1, *p*<0.01. Republicans also had significantly more “Wikipedia” boxes (M = 3.16, SD = 4.00) than “Party” boxes (M = 1.26, SD = 0.92), *t* = 3.5, *p*<0.001. Tables 2 and 3 provide descriptive statistics for the identity analysis.

**Table 2:** Average Number of Userboxes per user.

	Total n° of Boxes	Political Boxes		
		Conservative	Liberal	Other
Democrats	49.24 (±5.51)	0.08 (±0.06)	2.51 (±0.49)	2.20 (±0.46)
Republicans	42.90 (±7.56)	3.06 (±0.76)	0.27 (±0.09)	2.52 (±0.76)
Diff. significant?	No	*p*<0.001	*p*<0.001	No

Values in parenthesis indicate the corresponding standard error of the means. Bottom row indicates the outcome of a t-test (n = 50) for a significant difference between the mean values of the supporters of the two parties.

**Table 3:** Average Number of Party vs. Wikipedia Userboxes per user.

	Party Boxes	Wikipedia Boxes	difference significant?
Democrats	1.44 (±0.19)	4.70 (±0.52)	p<0.01
Republicans	1.26 (±0.13)	3.16 (±0.56)	p<0.001

Values in parenthesis indicate the corresponding standard error of the means. Last column indicates the outcome of a paired t-test (n = 50) for significant difference between the mean values of party and Wikipedia boxes within the supporters of the two parties.

### Most edited articles

Out of the 100 most edited articles, Democrats and Republicans had 44 articles in common. For Democrats, 38 of the top 100 most edited articles dealt with political topics. Out of those, 15 were coded as liberal, 15 were coded as conservative, and 8 were coded as neutral. Thirty-five out of the top 100 most edited articles by Republicans dealt with political topics. Of these, 7 were coded as liberal, 17 were coded as conservative, and 11 were coded as neutral. These findings stand in contrast to the most edited articles for users in general, only 22 of which dealt with political topics. Of those, 3 were coded as conservative, 5 were coded as liberal and 14 were coded as neutral. Table 4 contains an overview of the top 10 most edited articles by Democrats, Republicans, and Wikipedians in general.

**Table 4:** Top 10 Articles per number of distinct editors among Democrats, Republicans, and all users.

Rank	Democrats	Republicans	All Users
1.	**Barack Obama**	**George W. Bush**	**George W. Bush**
2.	**Unites States presidential election, 2008**	**Unites States presidential election, 2008**	Wikipedia
3.	**George W. Bush**	United States	United States
4.	Unites States	**Republican Party (United States)**	**Barack Obama**
5.	**Bill Clinton**	**John McCain**	Adolf Hitler
6.	**Democratic party (United States)**	**Barack Obama**	Michael Jackson
7.	Wikipedia	Wikipedia	Britney Spears
8.	Britney Spears	**Ronald Reagan**	Jesus
9.	**Hillary Rodham Clinton**	Virginia Tech Massacre	World War II
10.	**Al Gore**	Adolf Hitler	PlayStation 3

### Cross-party interactions

Tables 5 and 6 show the numbers of edges in the two networks under examination, broken down by party. The two tables show the values obtained for the unweighted networks and represent the numbers of pairs of users interacting. The values for the weighted networks are reported in parentheses; they account for the weight of each connection, and represent the total numbers of messages exchanged among different groups of users.

**Table 5:** Pairs of users interacting in article discussions, broken by party. In parentheses the total number of messages exchanged.

	Democrats		Republicans	
Democrats	193	(384)	94	(215)
Republicans	86	(180)	57	(75)

**Table 6:** Pair of users interacting on personal walls, broken by party. In parentheses the total number of messages exchanged.

	Democrats		Republicans	
Democrats	395	(1263)	243	(665)
Republicans	187	(519)	172	(738)

**Figure 5:**
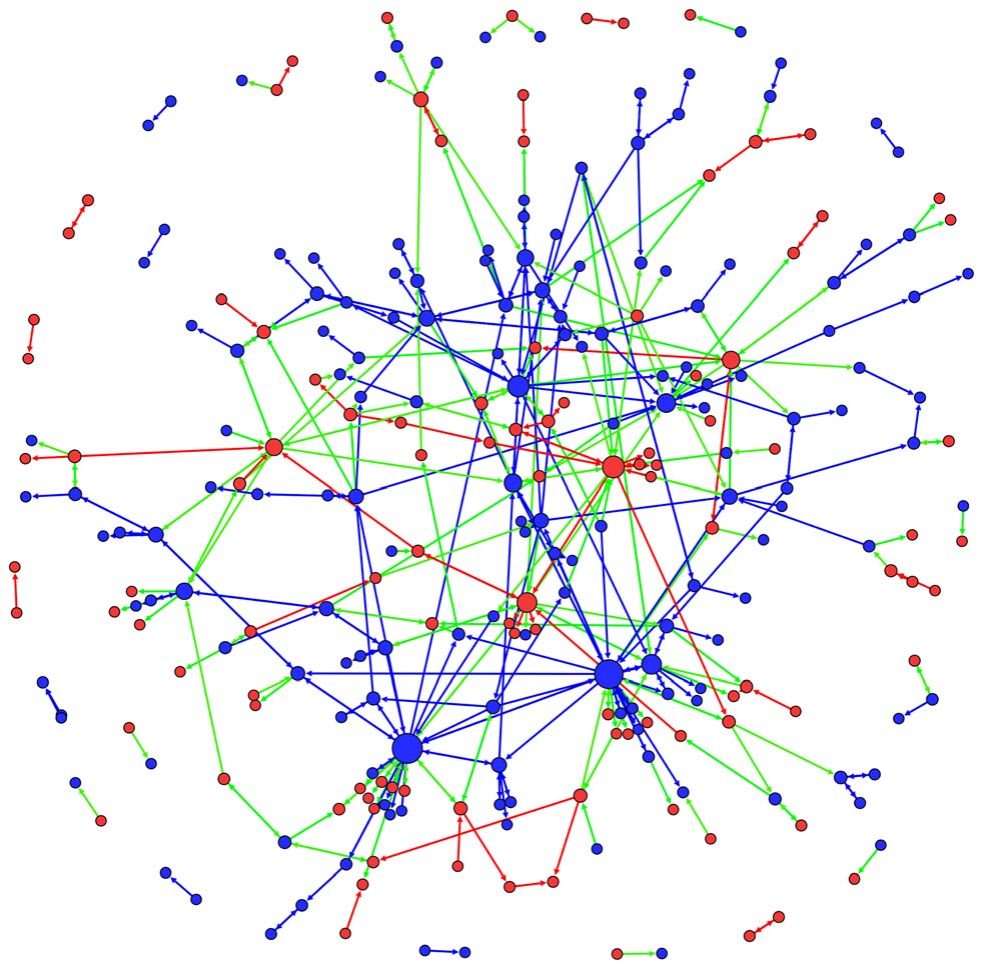
Reply network. Blue nodes represent Democrats, and red nodes Republicans. The size of each node is proportional to the number of connections (degree) in the unweighted reply network. Edges connecting two Democrats are depicted in blue, edges connecting two Republicans in red, edges connecting a Democrat and a Republican in green.

The results of a shuffle test for assortativity, shown in Table 7, indicate that users exhibit no significant preference either for interacting with same party or different party users on article talk pages (|Z|<2). That is, Democrats are not significantly more likely to interact either with other Democrats or with Republicans, nor are Republicans significantly more likely to interact either with other Republicans or with Democrats in the context of discussions on article talk pages. This result holds both in the weighted and in the unweighted networks, i.e. both considering the distinct pairs of users interacting, and the total numbers of messages exchanged among parties. Figure 5 helps to understand this finding, showing, for the unweighted network, intra-party connections in blue (between Democrats) and red (between Republicans), and inter-party connections in green: as can be observed, the number of inter-party connections is remarkable.

**Table 7:** Mixing by party in the interaction networks.

	r	r_rand_ (avg)	σ*_rand_*	Z-score
Reply (unweighted)	0.070	0.0028	0.0505	1.33
Reply (weighted)	−0.062	−0.0122	0.0316	−1.59
Wall (unweighted)	0.095	−0.0053	0.0301	**3.33**
Wall (weighted)	0.237	−0.0017	0.0131	**18.22**

r represents the mixing coefficient in the real network; r_rand_ (avg) and σ*_rand_* the average and the standard deviation of the mixing coefficient over the randomized networks; Z-score the standard score. Values indicating statistically significant results (Z>2) are written in bold.

An examination of the interactions on user walls shows a different pattern, indicating a significant preference for interaction among members of the same party (Z-score = 3.33 for the unweighted network and 18.22 for the weighted network, see Table 7) in this more personal communication space. The higher value for the weighted network seems to indicate that, beyond being more likely to interact with each other, users of the same party also tend to exchange more messages with one another than with members of the other party with whom they interact. That is, intra-party interactions in personal spaces are not only more likely to happen, but also more intense.

### Conflict analysis

Finally, we examined levels of conflict in discussion threads that dealt with political or other potentially controversial topics. There were relatively high levels of conflict across all three groups of threads that we examined. This is not a particularly surprising finding, given that we purposefully selected threads that dealt with controversial topics. Seventy-four percent of the threads that involved at least one Democrat and one Republican, 65% of threads involving at least two Democrats, and 77% of threads involving at least two Republicans were conflictive. See Table 8 for an overview. In order to assess differences in levels of conflict across the three groups we used a Chi-square goodness-of-fit, with the null hypothesis that the proportion of conflict is the same for the three subsets as in totality. There was a significantly (p<0.05) lower volume of conflict in the threads involving two Democrats, but there was no significant difference in the volume of conflict in the Republican (p = 0.45) and cross-party threads (p = 0.15).

**Table 8:** Number of politically valenced threads displaying conflict.

	Involving a Democrat and a Republican	Involving two Democrats	Involving two Republicans
Total number of threads	583	572	147
Political threads	144	130	71
Political threads displaying conflict	106	80	53

## Discussion

Our results paint an interesting and somewhat mixed picture of the nature of interactions among members of the Wikipedia community who espouse a political affiliation. First, we examined identity representation practices. We found that a subset of users on Wikipedia publicly proclaim their political affiliation through userboxes, and users who proclaim their affiliation for a particular party tend to have high numbers of userboxes that are ideologically aligned with that party. However, these ‘political’ users also had equally high numbers of Wikipedia userboxes. That is, boxes that espoused an identity of being a ‘Wikipedian’. The results indicate that the social identities of being a member of a political party and of being a Wikipedian may be equally important. Analysis of patterns of activity and interaction indicates that which identity is activated may depend both on user context and the nature of activities in which users are engaged.

An examination of the most edited articles for each group reveals that Democrats and Republicans both exhibit a tendency for editing articles that deal with political topics. For both groups, roughly one-third of the most edited articles dealt with political topics, compared to less than one-quarter for users in general. Interestingly, for both groups we find a preference for topics directly related to their party, such as “Barack Obama” or “Bill Clinton” for Democrats, and “John McCain” or “Republican Party” for Republicans.

Laniado, Castillo, Kaltenbrunner and Fuster-Morell [42] investigated another source of social identity, gender, and found that women preferentially edit certain topics, and are also more likely to interact with other female users than with men in discussions about the encyclopedic content (i.e. in article talk pages). In contrast, partisan users, despite their preference for working on articles in line with their party's ideology, did not exhibit a preference for interacting with other members of their same political party. We also did not observe a preference for inter-party interactions, as we might expect if there were a prevalence of partisan discussions, with most users acting as “fighters” [10] and engaging in disputes with users supporting the other party. Instead, we observe no significantly prevalent mixing pattern: when dealing with encyclopedic content, editors appear to be equally likely to engage conversations with users from the other party as with users from the same party.

However, we did see evidence for preference to interact with members of the same party in user walls. It is interesting that we observe this tendency in these more personal spaces, but not on article talk pages. It may be that in the course of conducting activities that are central to the Wikipedia community (e.g. editing articles), the identity of being a Wikipedian is activated and, as a result, the political identity is not salient. In the context of interactions on user walls, where personal activities take greater precedence, the importance of political ideology may shine through more strongly.

Finally, we found that levels of conflict were high both within and across parties when the discussion threads dealt with political or other potentially controversial topics. Interestingly, there were a significantly greater number of conflictive cross-party and Republican threads, indicating that Democrats have lower rates of within party conflict in the context of these controversial threads.

## Conclusions

Although Democrats and Republicans seem to maintain their political identity within the Wikipedia community, our findings show that users displayed more “Wikipedia” boxes than political boxes on their user pages, indicating that the identity of being a Wikipedian may be more salient in the context of this community. Further, the lack of preference to interact with same-party members in the context of article discussions does not indicate the same polarization that has been observed in other contexts [1], [8], [9]. In this sense, the Wikipedian identity seems to predominate over party identity. Hence, the results of our analysis show that despite the increasing political division of the U.S., there are still areas in which political dialogue is possible and happens.
